# PTPN2 deficiency along with activation of nuclear Akt predict endocrine resistance in breast cancer

**DOI:** 10.1007/s00432-018-2810-6

**Published:** 2018-12-04

**Authors:** Elin Karlsson, Cynthia Veenstra, Jon Gårsjö, Bo Nordenskjöld, Tommy Fornander, Olle Stål

**Affiliations:** 1 Department of Clinical and Experimental Medicine, Department of Oncology, Linköping University, 58185 Linköping, Sweden; 2 Department of Oncology, Karolinska University Hospital and Karolinska Institute, 17176 Stockholm, Sweden

**Keywords:** TCPTP, 18p deletion, PTPN2, Protein tyrosine phosphatase, Breast cancer, Gene copy number, Immunohistochemistry

## Abstract

**Purpose:**

The protein tyrosine phosphatase, non-receptor type 2 (PTNP2) regulates receptor tyrosine kinase signalling, preventing downstream activation of intracellular pathways like the PI3K/Akt pathway. The gene encoding the protein is located on chromosome 18p11; the 18p region is commonly deleted in breast cancer. In this study, we aimed to evaluate PTPN2 protein expression in a large breast cancer cohort, its possible associations to *PTPN2* gene copy loss, Akt activation, and the potential use as a clinical marker in breast cancer.

**Methods:**

PTPN2 protein expression was analysed by immunohistochemistry in 664 node-negative breast tumours from patients enrolled in a randomised tamoxifen trial. DNA was available for 146 patients, *PTPN2* gene copy number was determined by real-time PCR.

**Results:**

*PTPN2* gene loss was detected in 17.8% of the tumours. Low PTPN2 protein expression was associated with higher levels of nuclear-activated Akt (pAkt-n). Low PTPN2 as well as the combination variable low PTPN2/high pAkt-n could be used as predictive markers of poor tamoxifen response.

**Conclusion:**

PTPN2 negatively regulates Akt signalling and loss of PTPN2 protein along with increased pAkt-n is a new potential clinical marker of endocrine treatment efficacy, which may allow for further tailored patient therapies.

## Introduction

Anti-oestrogen treatment significantly reduces the recurrence and death rates in women with oestrogen receptor (ER)-positive breast cancer. Endocrine therapy is a well-tolerated treatment to which most ER-positive tumours respond, however, around 30% of the ER-positive tumours show de novo or acquired resistance to the treatment. A commonly suggested mechanism to this resistance is the crosstalk between ER and growth factor signalling pathways, specifically the receptor tyrosine kinase (RTK)/PI3K/Akt/mTOR axis (Musgrove and Sutherland 2009; Miller 2013). RTK signalling consists of complex networks of proteins with numerous feedback mechanisms. Protein tyrosine phosphatases (PTP) negatively regulate RTK signalling by dephosphorylation of tyrosine residues. Genetic and/or epigenetic alterations resulting in deregulation of PTP function have been shown to contribute to the development of several diseases, including cancer (Bussieres-Marmen et al. 2014; He et al. 2014; Julien et al. 2011).

One such PTP is protein tyrosine phosphatase, non-receptor 2 (PTPN2). PTPN2 was first found in T-cells and is, therefore, also known as T-cell PTP (TCPTP) (Mosinger et al. 1992). The gene encoding PTPN2 is located in the chromosomal region 18p. This region is commonly deleted in breast cancer and associated with poor outcome (Addou-Klouche et al. 2010; Climent et al. 2002; Karlsson et al. 2015). Alternative splicing produces two main isoforms, the original 48.5 kDa (TC48) and a 45 kDa (TC45) isoform. TC48 contains a hydrophobic C-terminus, mainly localising it to the endoplasmic reticulum. The shorter TC45 is primarily targeted to the nucleus in resting cells but can enter the cytoplasm upon growth factor stimuli (Tiganis 2013). PTPN2 is ubiquitously expressed and has been shown to regulate receptor tyrosine kinase signalling, thereby preventing downstream activation of intracellular pathways, amongst others the PI3K/Akt pathway (Klingler-Hoffmann et al. 2001; Tiganis et al. 1999). PTPN2-regulated receptors include the epidermal growth factor receptor (EGFR), the insulin receptor (IR), the vascular endothelial growth factor receptor (VEGFR) and Met (Galic et al. 2003, 2005; Mattila et al. 2008; Omerovic et al. 2010; Tiganis et al. 1998, 1999; Sangwan et al. 2008). Due to its involvement in the regulation of these oncoproteins, PTPN2 has been suggested to be a tumour suppressor.

This study aimed to evaluate PTPN2 protein expression in a large breast cancer patient material, its possible associations with *PTPN2* loss, Akt activation, and the potential use as a new clinical marker in breast cancer.

## Materials and methods

### Patient material

**Fig. 1:**
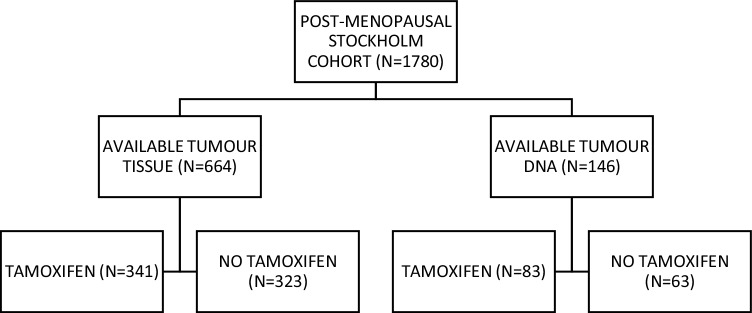
Patient flow through the randomised Stockholm tamoxifen trial

The cohort consisted of post-menopausal breast cancer patients enrolled in a randomised adjuvant trial between November 1976 and April 1990. Study design and long-term follow-up data have been previously reported in detail (Rutqvist et al. 2007). Briefly, breast cancer patients with a tumour diameter of ≤ 30 mm and no lymph node involvement were included in the cohort. The patients were randomised to receive post-operative tamoxifen for 2 years or no endocrine treatment. The women who were recurrence-free after 2 years of tamoxifen treatment and who consented were randomised to three additional years of tamoxifen or no further treatment. All patients were primarily treated with a modified radical mastectomy. Tumour tissues were formalin-fixed paraffin-embedded and stored at room temperature. Tumour tissue material in the form of tissue microarrays (TMA) was still available from 664 patients and high-quality DNA could be prepared from 146 patients (Fig. 1). Tumour characteristics and treatment were comparable with the original cohort (Bostner et al. 2010). The local ethics board at the Karolinska Institute, Stockholm, Sweden, approved retrospective studies of biomarkers.

### Clinicopathological variables and biomarkers

The status of ER and progesterone receptor (PR) were previously analysed by immunohistochemistry (IHC) (Jerevall et al. 2011). The cut-off level for both ER and PR positivity was > 10% stained nuclei. When IHC data was not available, ER status determined at the time of diagnosis was used with the cut-off of 0.05 fmol/µg DNA (Rutqvist et al. 2007; Rutqvist and Johansson 2006). Isoelectric focusing and IHC data have been shown to be comparable in this cohort (Khoshnoud et al. 2011). Nottingham histological grade and HER2 protein expression were evaluated retrospectively (Jansson et al. 2009; Jerevall et al. 2011). Staining and grading of Akt, phosphorylated at S473 (pAkt), have been described previously (Bostner et al. 2013). In this study, cytoplasmic pAkt staining is referred to as pAkt-cyt. pAkt expression that was stronger in the nucleus than in the cytoplasm is referred to as pAkt-n > cyt.

### Quantitative PCR

Total DNA was prepared from FFPE tissue using the QIAamp DNA FFPE Tissue Kit (Qiagen, Hilden, Germany). The breast cancer cell line MCF7 was used as a calibrator for the quantitative PCR. The cell line was purchased from the American Type Culture Collection (ATCC) in 2013 and authenticated by the company through STR analysis. The cells were subcultured for one passage upon arrival and tested negative for mycoplasma (LookOut® Mycoplasma Detection Kit, Sigma-Aldrich, St Louis, MO, USA). DNA for standardisation was prepared from the cells using the DNeasy Blood and Tissue Kit (Qiagen). For estimation of *PTPN2* gene deletion, fast real-time PCR was performed as previously described using an ABI Prism 7900ht (Applied Biosystems, Foster City, CA, USA) with the default thermal conditions: 95 °C for 20 s; 40 cycles of 95 °C for 1 s, and 60 °C for 20 s (Karlsson et al. 2015). Briefly, 10–25 ng total DNA was added to a 10 µL reaction with 1x Taq Man Fast Universal PCR master mix (Applied Biosystems) and 0.1 µM primer and probe for *PTPN2* or the endogenous control Amyloid Precursor Protein (APP). *PTPN2* gene quantification was performed with the Comparative Ct method using DNA from the cell line MCF7 as the calibrator sample on each plate. Samples were run in triplicates and standard deviations < 0.3 were required for inclusion in further analysis. With this criterion, *PTPN2* status was obtained for 146 patients. Primers and probes sequences were as follows: *PTPN2* forward primer: 5ʹ-AAGCCCACTCCGGAAACTAAA-3ʹ, *PTPN2* reverse primer: 5ʹ-AAACAAACAACTGTGAGGCAATCTA-3ʹ, *PTPN2* probe: 5ʹ-TGAGGCTCGCTAACC-3ʹ, *APP* forward primer: 5ʹ-TTTGTGTGCTCTCCCAGGTCT-3ʹ, APP reverse primer: 5ʹ-TGGTCACTGGTTGGTTGGC-3ʹ, APP probe: 5ʹCCCTGAACTGCAGATCACCAATGTGGTAG-3ʹ.

### Immunohistochemistry

**Fig. 2:**
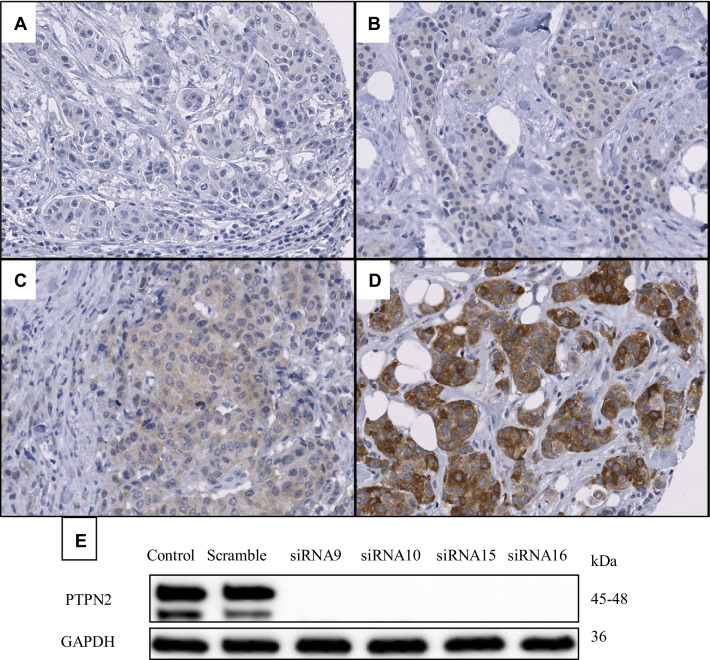
Representative images of PTPN2 protein staining: negative (**a**), weak (**b**), moderate (**c**), and strong (**d**). Validation of the antibody, which recognises both known isoforms of PTPN2, using four different siRNAs against PTPN2 is shown in panel (**e**)

Protein expression of PTPN2 in the available tumours was evaluated with immunohistochemistry. First, tissue microarrays (TMAs) were created; triplicates of core needle biopsies from paraffin-embedded tissues were re-embedded in new paraffin blocks. The blocks were cut into 4 µM sections and mounted on frost-coated slides. Deparaffinisation, rehydration and antigen retrieval of the slides was performed with the PT link system (Dako, Glostrup, Denmark) in Envision FLEX Target Retrieval Solution Low pH. Endogenous peroxidases were blocked with 3% H_2_O_2_ in H_2_O, for 10 min. To reduce unspecific binding, protein block X0909 (Dako) was applied for 10 min. The slides were incubated with PTPN2 primary antibody (Proteintech, Rosemont, IL, USA; 11214-1-AP, diluted 1:40) overnight at 4 °C. The secondary antibody (EnVision™, Dako) was applied for 30 min at room temperature. For visualisation, the Dako Liquid DAB + Substrate Chromogen System was used according to manufacturer’s instructions (Dako), where slides were incubated for 4 min with DAB:substrate buffer, 1:40. Counterstaining was performed with haematoxylin (Biocare Medical, Concord, CA, USA) for 30 s, in room temperature and darkness. Whole-slide images were generated with the Aperio ScanScope AT at 200x magnification (Leica Biosystems, Wetzlar, Germany) and staining was evaluated with the Imagescope software (Leica Biosystems). Two independent observers performed grading. PTPN2 was assessable in 664 tumours and cytoplasmic staining was graded as negative, weak, moderate or strong (Fig. 2a–d). These groups were dichotomised for further analyses into a low group, comprised of the negative and weak staining, and a high group including moderate and strong staining. Protein specificity of the PTPN2 antibody was validated with four different siRNAs against PTPN2, to wit: Hs_PTPN2_9 (siRNA9), Hs_PTPN2_10 (siRNA10), Hs_PTPN2_15 (siRNA15), Hs_PTPN2_16 (siRNA16) (Qiagen). MCF7 cells were transfected with 10 µM siRNA using Dharmafect 1 (Dharmacon, Thermo Fisher Scientific, Waltham, MA, USA) as a transfection agent and cells were incubated for 48 h with siRNA. Western blot was performed to visualise specificity (Fig. 2e).

### Statistical analysis

Spearman rank order correlation test was used to determine the association between *PTPN2* gene copy number and protein expression levels. The relationships between *PTPN2* gene copy number, protein expression, and clinical variables were assessed by the Chi-square test or Chi-square test for trend, when appropriate. The product-limit method was used for estimation of cumulative probabilities of distance recurrence-free survival (DRFS). Differences in survival between groups were tested with the log-rank test. Analysis of distant recurrence rates, as well as interaction tests, were performed with Cox proportional hazard regression. All the procedures were comprised in STATISTICA 12 (Statsoft, Inc, Tulsa, OK, USA). The criterion for statistical significance was *P* < 0.05.

## Results

The gene copy number status of *PTPN2* could be analysed in 146 available tumour samples, whereas PTPN2 protein expression could be assessed in 664 tumours. *PTPN2* gene deletion was detected in 17.8% (26/146) of the tumours. PTPN2 expression was found to be negative in 19.0% (126/664) of the cases and weak in 34.6% (228/664) of the cases. Grouped together they formed the PTPN2 low group, which in total accounted for 53.3% (354/664) of the cases. PTPN2 high (46.7%; 310/664) comprised of moderate (31.2%; 208/664) and strong staining (15.4%; 102/664). A trend to positive correlation between *PTPN2* gene fold change and protein expression was found (*P* = 0.088).

### Correlations to clinical variables and pAkt expression

The associations between PTPN2 and clinicopathological parameters were further assessed. PTPN2 protein expression correlated with ER positivity in the tumours (*P* = 0.0066, Table 1) and borderline associated with PR expression (*P* = 0.058). Furthermore, it was found to be correlated with pAkt-cyt (*P* < 0.0001, Table 1) and inversely correlated with pAkt-n > cyt (*P* = 0.006, Table 1). *PTPN2* gene deletion was not significantly associated to any of the clinical variables in the analysis.

**Table 1:** *PTPN2* gene copies and PTPN2 protein expression levels in relation to clinicopathological factors and Akt phosphorylation

	PTPN2 gene copies			PTPN2 protein		
	Deletion	Two or more copies	*p* value	Low	High	*p* value
	*n* (%)	*n* (%)		*n* (%)	*n* (%)	
All patients	26 (17.8)	120 (82.2)		354 (53.3)	310 (46.7)	
Tamoxifen treated						
No	11 (17.5)	52 (82.5)		175 (54.2)	148 (45.8)	
Yes	15 (18.1)	68 (81.9)	*P* = 0.92	179 (52.5)	162 (47.5)	*P* = 0.66
Tumour size						
≤ 20 mm	21 (19.3)	88 (80.7)		267 (53.2)	235 (46.8)	
> 20 mm	5 (13.5)	32 (86.5)	*P* = 0.43	84 (55.6)	67 (44.4)	*P* = 0.60
Nottingham grade						
1	1 (5.6)	17 (94.4)		57 (52.3)	52 (47.7)	
2	17 (22.7)	58 (77.3)		177 (54.5)	148 (45.5)	
3	8 (20.0)	32 (80.0)	*P* = 0.42	66 (45.8)	78 (54.2)	*P* = 0.23
ER						
Negative	5 (16.1)	26(83.9)		91 (63.2)	53 (36.8)	
Positive	21 (18.6)	92 (81.4)	*P* = 0.75	256 (50.4)	252 (49.6)	***P*** = **0.0066**
PgR						
Negative	14 (21.5)	51 (78.5)		160 (56.1)	125 (43.9)	
Positive	12 (15.8)	64 (84.2)	*P* = 0.38	158 (48.5)	168 (51.5)	*P* = 0.058
HER2 protein						
Negative	23 (19.5)	95 (80.5)		291 (52.0)	269 (48.0)	
Positive	3 (20.0)	12 (80.0)	*P* = 0.96	37 (52.1)	34 (47.9)	*P* = 0.98
pAkt–cytoplasm						
−	9 (24.3)	28 (75.7)		171 (66.8)	85 (33.2)	
+	14 (14.6)	82 (85.4)	*P* = 0.18	172 (44.0)	219 (56.0)	***P*** ≤ **0.0001**
pAkt-n > cyt						
No	17 (17.9)	78 (82.1)		206 (49.1)	214 (51.0)	
Yes	6 (15.8)	32 (84.2)	*P* = 0.77	137 (60.4)	90 (39.7)	***P*** = **0.0060**

*P*-values printed in bold are considered significant

### PTPN2 in relation to prognosis

The rate of distant recurrences was similar for systemically untreated patients with high-expressing PTPN2 tumours as well as low-expressing (high vs low, HR = 1.22, 95% CI 0.80–1.86, *P* = 0.37). Dividing tumours by their Nottingham grade (NHG), a trend was found where low PTPN2 expression indicated a higher risk for distant recurrence in NHG 1 tumours (HR = 0.41, 95% CI0.12–1.42, *P* = 0.16) compared with NHG 2–3 tumours (HR = 1.34, 95% CI 0.83–2.15, *P* = 0.23).

### PTPN2 and nuclear pAkt predict tamoxifen benefit

**Fig. 3:**
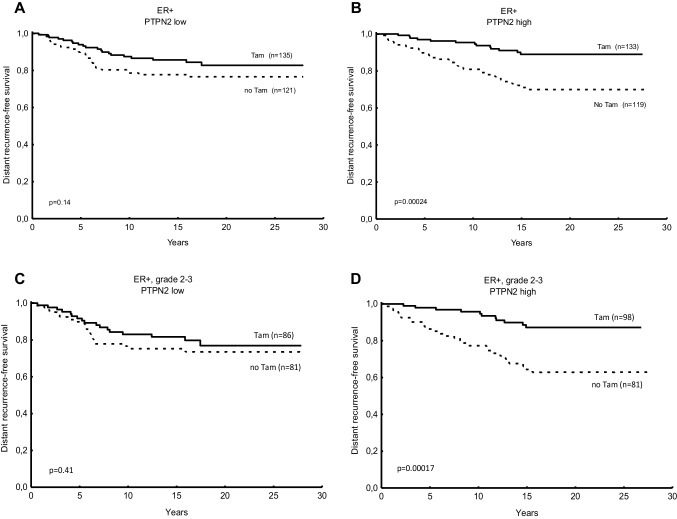
Predictive value for tamoxifen benefit of PTPN2 protein expression. Distant recurrence-free survival (DRFS) for breast cancer patients treated with tamoxifen (Tam) vs no tamoxifen in relation low PTPN2 protein in oestrogen receptor-positive (ER+) tumours (**a**), high PTPN2 protein expression in ER + tumours (**b**), low PTPN2 protein expression in ER + tumours with grade 2 or 3 (**c**), and high PTPN2 protein expression in ER + tumours with grade 2 or 3 (**d**). *P* values were estimated with the log rank test

Patients with tumours expressing low levels of PTPN2 had no significant benefit from tamoxifen treatment (*P* = 0.14), whereas the group with high protein expression did have benefit (*P* = 0.00005, interaction test *P* = 0.11; Table 2; Fig. 3a, b). Restricted to patients with grade 2 or 3 tumours, the interaction test reached significance (*P* = 0.043, Table 2; Fig. 3c, d).

**Table 2:** Cox proportional hazard regression of distant recurrence rate for patients treated with adjuvant tamoxifen vs no tamoxifen, in relation to PTPN2 protein expression and the expression of PTPN2 protein and nuclear pAkt expression in combination. *P* values printed in bold are considered significant

	No. of patients	Tamoxifen vs no tamoxifen HR (95% CI)	*P* value for interaction	
ER +				
PTPN2 low	256	0.65 (0.36–1.15) *P* = 0.14	*P* = 0.11	(Figure 3a)
PTPN2 high	252	0.31 (0.17–0.61) *P* = **0.0005**		(Figure 3b)
PTPN2 low or pAkt-n high	356	0.64 (0.39–1.05) *P* = 0.077	*P* = **0.044**	(Figure 4a)
PTPN2 high and pAkt-n low	168	0.23 (0.10–0.54) ***P*** = **0.0007**		(Figure 4b)
ER + and NHG 2–3				
PTPN2 low	167	0.76 (0.40–1.46) *P* = 0.41	*P* = **0.043**	(Figure 3c)
PTPN2 high	179	0.28 (0.14–0.57) ***P*** = **0.0004**		(Figure 3d)
PTPN2 low or pAkt-n high	233	0.71 (0.41–1.23) *P* = 0.22	*P* = **0.019**	(Figure 4c)
PTPN2 high and pAkt-n low	121	0.18 (0.068–0.48) ***P*** = **0.0006**		(Figure 4d)

*P*-values printed in bold are considered significant

**Fig. 4:**
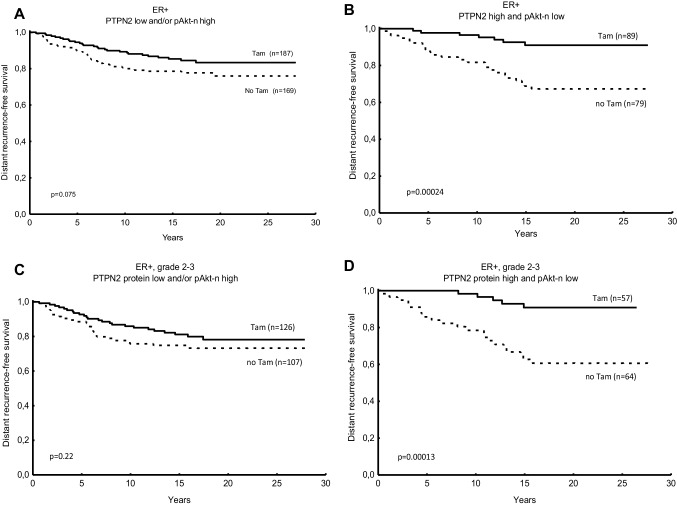
Predictive value for tamoxifen response of PTPN2 protein expression and nuclear phosphorylated Akt (pAkt-n) expression. Distant recurrence-free survival (DRFS) for breast cancer patients treated with tamoxifen (Tam) vs no tamoxifen in relation to low PTPN2 protein expression and high pAkt-n expression (oestrogen receptor-positive (ER+), (**a)**, high PTPN2 protein expression and/or pAkt-n expression low in ER + tumours (**b**), PTPN2 expression low and pAkt-n high in ER + tumours with grade 2 or 3 (**c**), and PTPN2 expression high and pAkt-n low in ER + tumours, grade 2 to 3 (**d**). *P* values were estimated with the log rank test

We previously showed that pAkt-n was borderline significant as a predictive factor of tamoxifen response in this cohort (Bostner et al. 2013). Because of the implications of PTPN2 as a regulator of Akt signalling, a combination variable was created including PTPN2 and nuclear pAkt protein levels. Tamoxifen treatment was associated with a strongly reduced risk of distant recurrence in the group of patients with ER-positive tumour and high PTPN2 concurrent with low pAkt expression, whereas no significant benefit from tamoxifen could be seen in the group with low PTPN2 and/or high nuclear pAkt (interaction test *P* = 0.044, Table 2; Fig. 4a, b). This predictive value of PTPN2 and nuclear pAkt was also evident when restricting the analysis to the group of patients with histologically grade 2–3 tumours (interaction test *P* = 0.019, Table 2; Fig. 4c, d).

## Discussion

Few studies have explored the role of PTPN2 in breast cancer; therefore, we aimed to evaluate the clinical value of PTPN2 in a large breast cancer cohort.

*PTPN2* gene copy loss could be detected in 17.8% of the cases, which is in agreement with our previous study on a post-menopausal breast cancer cohort (Karlsson et al. 2015). Low PTPN2 protein expression was detected in 53.3% of the cases. We found a trend to correlation between *PTPN2* gene deletion and expression levels of the corresponding protein. While, to our knowledge, there are no studies looking at the correlation between gene deletion and protein expressions levels, *PTPN2* gene deletion has previously been associated with low corresponding mRNA levels (Addou-Klouche et al. 2010; Karlsson et al. 2015). Whether genomic loss of *PTPN2* leads to decreased expression of its corresponding protein is still unclear.

Like previous studies, loss of PTPN2 was most common in the ER-negative subgroup (Shields et al. 2013; Karlsson et al. 2015). Low PTPN2 protein expression was associated with poor response to tamoxifen in the group of patients with tumours histologically graded as 2 or 3, suggesting a need for other types of treatment in this group. Patients with NHG 1 tumours with low PTPN2 tended to have a higher risk for distant recurrence, indicating that PTPN2 loss might have a prognostic value in patients with NHG1, which is normally associated with good prognosis.

We previously found a correlation between *PTPN2* gene deletion and high levels of phosphorylated Akt in breast cancer patients (Karlsson et al. 2015). Lee and colleagues showed significantly higher levels of phosphorylated Akt in PTPN2 knockout mice (Lee et al. 2017). In the present study, we provide further indications that PTPN2 regulates Akt signalling by showing that low PTPN2 protein expression was associated with increased nuclear pAkt levels. Increased levels of phosphorylated Akt in the nucleus have been shown to be associated with poor response to tamoxifen in breast cancer patients (Bostner et al. 2013) and the oestrogen receptor has been shown in vitro to be a direct substrate of Akt phosphorylation (Campbell et al. 2001). Interestingly, Akt activation and translocation to the nucleus have been shown to be promoted by the oncogene T-cell leukaemia/lymphoma 1B (TCL1), in turn, activated by oestrogen signalling (Pekarsky et al. 2000; Badve et al. 2010). When low PTPN2 protein was analysed in combination with high nuclear expression of phosphorylated Akt, the combination variable was a strong predictor of tamoxifen resistance amongst all patients with ER-positive breast cancer.

In summary, this study demonstrates that PTPN2 negatively regulates Akt signalling and that loss of PTPN2 protein along with increased nuclear pAkt may be a new potential clinical marker of endocrine treatment benefit, which may allow for further tailored patient therapies.
